# Comparison of the Clinical Manifestations of Acute Coronary Syndrome Between Diabetic and Non-diabetic Patients: A Systematic Review and Meta-Analysis

**DOI:** 10.7759/cureus.64311

**Published:** 2024-07-11

**Authors:** Godfrey Tabowei, Samuel K Dadzie, Saeed Ahmed, Muskan Lohana, Marium Shahzad, Syeda Nosheen Zehra, Mahnoor Zubair, Areeba Khan

**Affiliations:** 1 Internal Medicine, Texas Tech University Health Sciences Center, Odessa, USA; 2 Internal Medicine, Piedmont Athens Regional Medical Center, Athens, USA; 3 Cardiology, Mohtarma Benazir Bhutto Shaheed Medical College, Mirpur, PAK; 4 Medicine, Liaquat University of Medical and Health Sciences, Hyderabad, PAK; 5 Medicine, Sialkot Medical College, Sialkot, PAK; 6 Medicine, Liaquat Medical College, Karachi, PAK; 7 Critical Care Medicine, United Medical and Dental College, Karachi, PAK

**Keywords:** systematic review and meta-analysis, chest pain, clinical manifestation, acute coronary syndrome, diabetes

## Abstract

The presentation of acute coronary syndrome (ACS) in patients with type 2 diabetes mellitus (T2DM) may differ from that of non-diabetic patients, potentially leading to delayed diagnosis and treatment. This meta-analysis aimed to compare the clinical presentation of ACS between diabetic and non-diabetic patients. A systematic search of PubMed, Excerpta Medica database (EMBASE), and Web of Science databases was conducted for observational studies published from January 2010 onwards. Studies comparing ACS symptoms between diabetic and non-diabetic patients were included. The odds ratio (OR) with 95% confidence intervals (CI) was calculated using a random-effects model. Eight studies with a total of 29,503 patients (23.03% diabetic) were included. Diabetic patients were significantly less likely to present with chest pain compared to non-diabetic patients (OR: 0.43, 95% CI: 0.30 to 0.63, p<0.001). Anxiety (OR: 2.20, 95% CI: 1.17-4.14), shortness of breath (OR: 1.49, 95% CI: 1.11-2.01), and neck pain (OR: 1.62, 95% CI: 1.03-2.54) were significantly more common in diabetic patients. Sweating/cold sweat was less common in diabetics (OR: 0.60, 95% CI: 0.34-1.07), though not statistically significant. Other symptoms showed minimal differences between groups. High heterogeneity was observed across studies for most symptoms. This meta-analysis demonstrates that diabetic patients with ACS are less likely to experience typical chest pain and more likely to present with atypical symptoms such as anxiety, shortness of breath, and neck pain. These findings emphasize the need for healthcare providers to maintain high vigilance for atypical ACS presentations in diabetic patients. Tailored diagnostic approaches, modified triage protocols, and enhanced patient education are crucial to improving the timely diagnosis and treatment of ACS in this high-risk population.

## Introduction and background

Type 2 diabetes mellitus (T2DM) is among the most prevalent chronic non-communicable diseases, with its global incidence rising significantly. In 2017, T2DM affected 8.8% of the adult population worldwide, and this figure is projected to climb to 9.9% by 2045 [1-2]. The growing number of T2DM cases and associated complications are substantially impacting the quality of life and have emerged as a major global public health concern [3]. Additionally, the occurrence of painless acute coronary syndrome (ACS) is higher in diabetic patients, often leading to delays in treatment [4]. 

Acute coronary syndromes, which encompass ST-elevation myocardial infarction (STEMI), non-ST elevation myocardial infarction (NSTEMI), and unstable angina, are major contributors to increased morbidity and mortality in individuals with diabetes mellitus (DM). These patients face a poorer prognosis following a myocardial infarction (MI) compared to those without DM [5]. The heightened risk of MI in diabetic populations may be attributed to factors such as lifestyle behaviors, hyperglycemia, hypertension, dyslipidemia, impaired renal function, and other vascular diseases, including cerebrovascular and peripheral arterial diseases [6]. Given the elevated cardiovascular risk and worse outcomes, it is crucial to understand the factors that contribute to this disparity in outcomes. 

It is believed that the clinical presentation of ACS differs between diabetic and non-diabetic patients. Chest pain is the main symptom of ACS, aiding early detection and commonly used to assess treatment effectiveness. While ECG can detect symptomatic ST changes, many individuals with severe coronary artery lesions might not experience angina pectoris. Besides chest pain, ACS can manifest with atypical symptoms such as mild back pain, unusual fatigue, nausea, vomiting, shortness of breath, fainting, palpitations, numbness in the hands, and indigestion [7]. Various factors may cause patients with ACS to present only atypical symptoms, making transient myocardial ischemia silent in these cases [4]. Patients whose initial symptoms do not include chest pain are at a higher risk of delayed medical attention and often receive less aggressive treatment, leading to higher hospital mortality rates [8]. 

Conducting a meta-analysis to compare the presentation of ACS between patients with and without diabetes mellitus is crucial. Several studies have suggested that diabetic patients may present with atypical symptoms or silent ischemia during an acute coronary event, leading to delayed recognition and treatment. However, individual studies have reported inconsistent findings regarding the difference in presentation between these two groups. A meta-analysis would help quantify the extent of this difference, reconcile the inconsistencies across studies, and provide a more precise estimate of the risk associated with atypical presentation in diabetic patients. This could ultimately inform clinical decision-making and improve outcomes for this high-risk population. 

## Review

Methodology 

Literature Search 

Electronic databases (PubMed, Excerpta Medica database (EMBASE), and Web of Science) were systematically searched for relevant studies published from January 2010 to the present date (2024), using a combination of MeSH terms and keywords related to "diabetes mellitus," "acute coronary syndrome," "myocardial infarction," "presentation," "symptoms," and "clinical manifestations." Additionally, reference lists of included studies and relevant review articles were manually searched for potentially eligible studies. The search was performed independently by two authors, and any disagreement between the two authors was resolved through discussion or consensus with the principal investigator. 

Study Selection 

Observational studies (cohort, case-control, or cross-sectional) comparing the clinical presentation of ACS between diabetic and non-diabetic patients were considered for inclusion. Studies had to report the proportion of patients presenting with atypical symptoms in both groups. We excluded studies that included patients other than ACS. Reviews, editorials, case reports, and case series were not included in the meta-analysis. The study selection process was performed by two independent authors. First, duplicate records were removed from the combined search results. Then, the titles and abstracts of the remaining records were screened to identify potentially eligible studies. The full texts of these studies were retrieved and assessed against the inclusion and exclusion criteria. Any disagreements during the study selection process were resolved through discussion and consensus between the two authors. 

Data Extraction 

Two independent reviewers extracted data from eligible studies using a standardized form. The extracted data included study characteristics (author, year, design, setting), participant characteristics (age, sex, diabetes status, hypertension), and outcomes of interest. Any disagreements that arose during the data extraction process were resolved through consensus or consultation with a third reviewer. 

Data Analysis Plan 

Review Manager (RevMan) 5.3 software package (The Cochrane Collaboration, London, UK) was used for the meta-analysis. The odds ratio (OR) with 95% confidence intervals (CIs) was calculated as the effect measure to compare the odds of presenting symptoms of ACS between diabetic and non-diabetic patients. A p-value less than 0.05 was considered significant. A random-effects model was used to pool the study-specific ORs to deal with variation among the study results. We compared the baseline characteristics between diabetic and non-diabetic individuals using OR for categorical variables and mean difference (MD) for continuous variables. Heterogeneity across studies was assessed using the I-squared statistic, with values of 25%, 50%, and 75% representing low, moderate, and high heterogeneity, respectively. 

Results 

**Figure 1:**
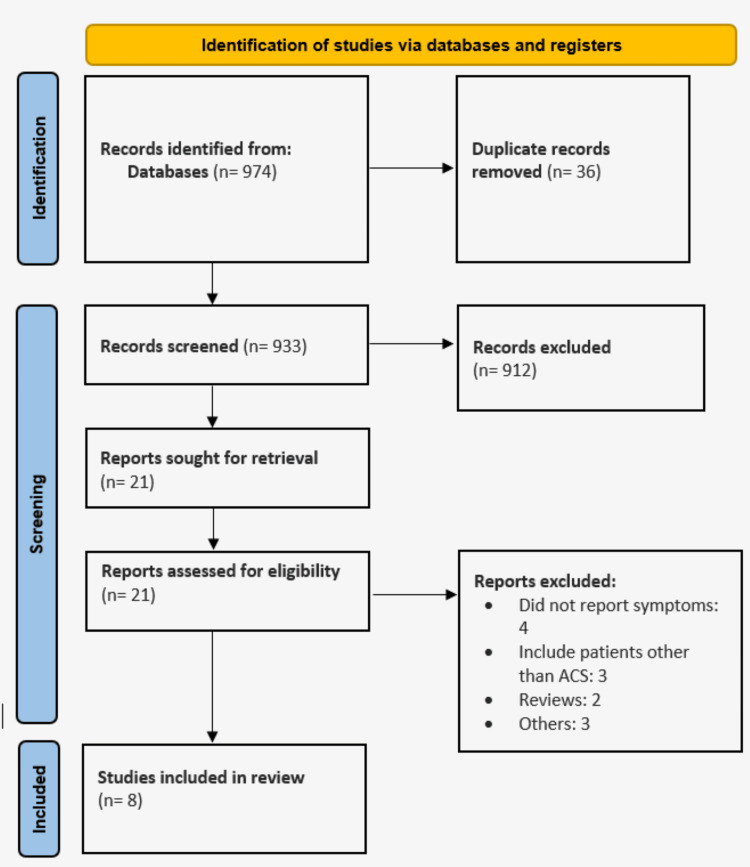
A PRISMA flowchart outlining the study selection process PRISMA: Preferred Reporting Items for Systematic Reviews and Meta-Analyses; ACS: acute coronary syndrome

Database searches yielded a total of 974 references. After removing duplicates, 933 records were screened using a title and abstract screening tool, resulting in 21 studies for which full-text reports were evaluated for eligibility. Of these, eight studies met the criteria for inclusion in the review. Figure 1 illustrates the study selection process, while Table 1 details the characteristics of the included studies. The combined sample size was 29,503, with 23.03% (n = 6,794) of the patients having diabetes. Table 2 compares the demographic characteristics of patients with and without diabetes. As shown, the mean age of diabetic patients was significantly higher compared to non-diabetic patients, although the number of males did not differ significantly between the two groups. Additionally, the prevalence of hypertension was significantly higher among diabetic patients with ACS compared to their non-diabetic counterparts.

**Table 1:** Characteristics of the included studies

Study ID	Region	Total population	Diabetes (n)	Without diabetes (n)
Ahmed et al., 2018 [9]	Pakistan	280	130	150
Angerud et al., 2016 [10]	Sweden	694	96	598
Dhanya et al., 2019 [11]	India	120	60	60
Fu et al., 2019 [12]	China	21,994	4,450	17,544
Manistamara et al., 2021 [13]	Indonesia	61	33	28
Paim et al., 2012 [14]	Portugal	88	11	77
Schmitz et al., 2024 [15]	Mexico	5,900	1,859	4,041
Taghipour et al., 2014 [16]	Iran	366	155	211

**Table 2:** Comparison of the characteristics of diabetic and non-diabetic individuals presenting with ACS OR: odds ratio; CI: confidence interval; ACS: acute coronary syndrome Presented as mean difference (95% CI)

Variables	OR (95% CI)	P-value
Age	2.02 (1.20 to 2.84)*	<0..001
Gender (males)	0.81 (0.65 to 1.02)	0.07
Hypertension	2.47 (1.62 to 3.79)	<0.001

Comparison of ACS Symptoms Between the Two Groups 

**Figure 2:**
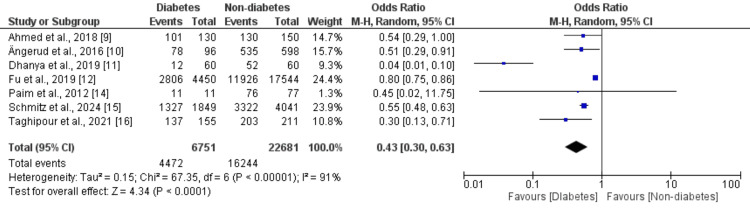
A comparison of chest pain as a symptom of ACS between patients with and without diabetes Sources: [9-12, 14-16]

We compared chest pain as a symptom of ACS between patients with and without diabetes, and the results are shown in Figure 2. The proportion of patients experiencing chest pain was significantly lower in patients with diabetes (n = 4,472) compared to patients without diabetes (n = 16,244) (OR: 0.43, 95% CI: 0.30 to 0.63, p-value<0.001). High heterogeneity was reported among the study results (I-square: 91%). 

Based on the findings in Table 3, several symptoms showed notable differences between diabetic and non-diabetic patients. Anxiety was significantly more common in diabetic patients (OR 2.20, CI 1.17-4.14), as was shortness of breath (OR 1.49, CI 1.11-2.01) and neck pain (OR 1.62, CI 1.03-2.54). Weakness and arm pain were more prevalent in diabetics, though not statistically significant. Conversely, sweating/cold sweat was less common in diabetics (OR 0.60, CI 0.34-1.07), as was palpitation, though with a wide confidence interval. Symptoms like nausea/vomiting, abdominal pain, jaw pain, and radiating pain showed minimal differences between groups. High I-square values for many symptoms indicated substantial heterogeneity across studies, and that could be because of differences in study region and sample size. These results suggest that symptom presentation may differ between diabetic and non-diabetic patients in certain aspects. 

**Table 3:** Comparison of the symptoms of ACS in patients with diabetes and those without diabetes. OR: odds ratio; CI: confidence interval; ACS: acute coronary syndrome

Symptoms	Diabetic	Non-diabetic	OR (95% CI)	I-square
Nausea/vomiting	20.48	23.21	0.86 (0.53 to 1.41)	86%
Shortness of breath	34.23	27.16	1.49 (1.11 to 2.01)	90%
Abdomen pain	5.32	4.14	1.00 (0.83 to 1.21)	25%
Sweating/cold sweat	41.20	47.19	0.60 (0.34 to 1.07)	90%
Jaw pain	6.74	5.36	0.87 (0.69 to 1.10)	41%
Neck pain	27.33	19.45	1.62 (1.03 to 2.54)	30%
Anxiety	36.51	17.37	2.20 (1.17 to 4.14)	78%
Syncope	4.34	3.40	1.42 (0.88 to 2.30)	85%
Weakness	45.82	37.21	1.46 (0.66 to 3.21)	85%
Radiating pain	30.60	32.12	0.94 (0.87 to 1.01)	0%
Palpitation	12.59	13.78	0.27 (0.02 to 3.25)	97%
Shoulder pain	18.65	16.98	1.28 (0.87 to 1.87)	38%
Arm pain	22.18	20.42	1.69 (0.68 to 2.01)	44%

Discussion 

This meta-analysis aimed to compare the presenting symptoms of patients with diabetes to those without diabetes. The study found that chest pain is less common in diabetic patients compared to non-diabetic patients. Additionally, symptoms such as anxiety and neck pain were significantly more prevalent in diabetic patients. Kumar et al. [17] conducted a meta-analysis, which revealed that among patients diagnosed with MI, those with diabetes were less likely to present with chest pain compared to non-diabetic individuals. 

Chest pain is a common symptom of ACS. However, previous studies have shown that patients with diabetes often experience less chest pain, making ACS diagnosis more challenging in this population [8, 18]. Chest pain is less common as a presentation of ACS in the diabetic population, primarily because of diabetic neuropathy. Diabetic neuropathy is a complication of diabetes that damages the nerves, including those that transmit pain signals. This nerve damage can dull or alter the sensation of pain, leading to atypical symptoms or even a complete absence of chest pain during cardiac events. Consequently, diabetic patients may not experience the typical chest pain associated with ACS, making it more challenging to recognize and diagnose heart attacks in this group [13, 19]. 

Chest pain is the primary warning sign of ACS, alerting both patients and physicians to potential underlying coronary artery disease. The absence of chest pain can significantly impact management by causing delays in identification, diagnosis, and treatment. The high prevalence of silent acute coronary events without chest pain among diabetic patients should prompt healthcare providers to be vigilant. Healthcare providers play a crucial role in preventing ACS and related comorbidities in diabetic and other high-risk patients [20-21]. Early detection of ACS in diabetic patients based on atypical symptoms is essential for initiating immediate intervention. To ensure timely hospital transfer, healthcare providers must educate diabetic patients and their caregivers about the symptoms of ACS, with a particular focus on atypical presentations [22]. However, more evidence from large cohorts of both diabetic and non-diabetic patients is needed to compare ACS symptomatology and identify specific atypical symptoms associated with diabetes. 

Additionally, diabetic patients may experience more pronounced autonomic symptoms, such as anxiety and shortness of breath, during an ACS event. This is also evident in our meta-analysis, which demonstrated significantly higher odds of dyspnea and anxiety in patients with shortness of breath [14]. The onset of symptoms in diabetics tends to be more gradual compared to the sudden, severe onset typically seen in non-diabetics, making it harder for patients and healthcare providers to recognize the urgency of the situation [23]. 

Gender differences add another layer of complexity, with women, especially those with diabetes, more likely to present with atypical symptoms. This can include back or jaw pain, nausea, or extreme fatigue without the classic chest pain [24]. Age also plays a role, as elderly patients in both groups may have atypical presentations, but this tendency is more pronounced in diabetics. The rationale behind these differences lies in the long-term effects of diabetes on the cardiovascular system, including accelerated atherosclerosis, endothelial dysfunction, and neuropathy [25]. These factors collectively alter the way the body perceives and responds to cardiac ischemia, resulting in a more varied and often subtle symptom profile in diabetic patients. Understanding these differences is crucial for healthcare providers to ensure timely diagnosis and treatment of ACS in diabetic patients, who are at higher risk for adverse outcomes due to their underlying metabolic condition [14]. 

The clinical implications of this meta-analysis underscore the need for a tailored approach to ACS management in diabetic patients. Clinicians must maintain heightened vigilance for atypical presentations, modify triage protocols, and expand diagnostic criteria. Patient education about varied symptoms is crucial. Lower thresholds for cardiac investigations and gender-specific approaches are necessary, especially for women and the elderly. These findings call for updated guidelines, refined diagnostic tools, and closer collaboration between endocrinologists and cardiologists to improve outcomes in this high-risk population. 

Research Implications 

Research implications from this meta-analysis highlight the need for gender-specific studies on ACS presentation in diabetic and non-diabetic populations. Priorities include investigating novel biomarkers, exploring pathophysiological mechanisms, developing tailored risk stratification models, and assessing the diagnostic accuracy of current tools. Studies should focus on treatment responses, long-term outcomes, and the potential of artificial intelligence (AI) in improving ACS detection. Genetic and epigenetic factors contributing to presentation differences warrant investigation. This research could significantly enhance diagnostic accuracy and treatment outcomes across all patient groups. 

Limitations 

Our results exhibited significant heterogeneity, likely due to the inclusion of diverse populations varying in diabetes type, MI categories, age, ethnicity, and other factors. The non-randomized studies in our analysis are expected to be more heterogeneous due to methodological and population differences compared to randomized trials. Although a subgroup analysis would be ideal to address this, the studies did not consistently report these variables. Moreover, we did not compare ECG changes between the two groups due to the unavailability of data.

Despite the underrepresentation of atypical presentations, our analysis focuses on a population already diagnosed with MI, indicating sufficient evidence for diagnostic testing from their initial presentation. Clinicians may have a higher suspicion of cardiovascular disease (CVD) in diabetic patients, who tend to access clinical care more frequently and are more easily admitted to hospitals, leading to more frequent investigations. Additionally, diabetic patients might have a stronger suspicion of MI, increasing their health-seeking behavior and the likelihood of receiving a diagnosis for MI. 

## Conclusions

Based on this meta-analysis, diabetic patients with ACS present differently than non-diabetic patients. They are less likely to experience chest pain, the classic ACS symptom, but more likely to have anxiety, shortness of breath, and neck pain. This atypical presentation can lead to delayed diagnosis and treatment, potentially worsening outcomes. These findings highlight the need for tailored approaches to diagnosing and managing ACS in diabetic patients, including modified triage protocols, expanded diagnostic criteria, and enhanced patient education. Healthcare providers must maintain high vigilance for atypical presentations in this high-risk population to ensure timely intervention and improved outcomes.
